# A systematic review of the effectiveness of self-symptoms monitoring with Patient Reported Outcome Measures in rheumatic disease patients

**DOI:** 10.1371/journal.pone.0338935

**Published:** 2025-12-30

**Authors:** Yu Heng Kwan, Livia Oh, Pui Kim Ang, Zhonghui Xiong, Pei Xin Chong, Sungwon Yoon, Pei Shi Ong, Charmaine T. M. Wang, Silvana X. Choo, Ying Ying Leung, Julian Thumboo, Warren Fong

**Affiliations:** 1 Program in Health Services and Systems Research, Duke-NUS Medical School, Singapore, Singapore; 2 Department of Rheumatology and Immunology, Singapore General Hospital, Singapore, Singapore; 3 Medicine Academic Clinical Program, Duke-NUS Medical School, Singapore, Singapore; 4 Department of Pharmacy, National University of Singapore, Singapore, Singapore; 5 Department of Occupational Therapy, Singapore General Hospital, Singapore, Singapore; CHU Nantes: Centre Hospitalier Universitaire de Nantes, FRANCE

## Abstract

**Objective:**

We aimed to provide an up-to-date synthesis of the effectiveness of patient reported outcome measures (PROMs) to self-monitor symptoms compared to conventional follow-up care in rheumatic disease patients. The effect of providing feedback via PROMs was also evaluated.

**Methods:**

This review was guided by the Preferred Reporting Items for Systematic review and Meta-Analysis guidelines. Articles published before December 2024 were retrieved from PubMed^®^, Cochrane Library^®^, Embase^®^, and PsycINFO^®^ (Ovid). Studies were included if they (1) compared PROMs against no PROMs use, or (2) or if they utilized PROMs as an intervention with feedback provided to healthcare professionals or patients for comparison against PROMs use without feedback. Non-English articles and abstract-only articles were excluded. Results were synthesized in a narrative manner. Methodological quality was assessed using the Risk of Bias tool and the Risk of Bias in Non-randomized Studies of Interventions tool.

**Results:**

A total of 18, 159 articles were screened, and 9 articles were included. All 9 studies reported on the use of PROMs as an intervention against a control where no PROMs were used. 4 of the studies included reported improvements in symptom control; 1 study observed improvement in health-related quality of life (HRQoL) when PROMs were used. High patient satisfaction was observed in 5 studies, but the results were statistically insignificant. 1 found that PROMs use facilitated shared decision making. 3 studies reported on clinic visits with mixed results. No studies reported on survival/mortality. Out of the 9 studies, 2 studies compared PROMs with feedback to patients and/or healthcare professionals against PROMS without feedback. There was conflicting evidence whether PROMs with feedback improved patient satisfaction in clinical care. Three studies were identified to be of moderate to high risk of bias.

**Conclusion:**

The use of PROMs self-symptom monitoring may contribute to improving symptom control, HRQoL, patient perception, promote shared decision making, and reduce clinic visits. Our study may have limited generalizability to other rheumatic disease beyond RA as most of our study is in rheumatoid arthritis. More studies in other rheumatic diseases are needed.

**Registration:**

The protocol was registered in OpenSci Framework (https://doi.org/10.17605/OSF.IO/ZU9XM).

## Introduction

Rheumatic diseases are one of the most common chronic diseases worldwide [1]. Some examples include rheumatoid arthritis, spondylarthritis and psoriatic arthritis. The goals of treatment in rheumatic diseases are to achieve remission or low disease activity [1]. Therefore, symptom monitoring is crucial as it is a cornerstone for management of rheumatic diseases [2,3]. Usual clinical care encompasses clinicians conducting a detailed history of symptoms patients are experiencing, which can be time-consuming. Patients may also find it difficult to accurately articulate their symptoms during visits, which can lead to suboptimal treatment decisions and poor disease control [4]. Frequent symptom monitoring also requires additional follow-up visits. However, long waiting times can be cumbersome and reduce patient compliance to follow-up visits. For example, a study in Malaysia reported an average waiting time of 1–2 hours for a consultation of only 10–15 minutes [5]. In patients with well-controlled symptoms, follow-up visits can be reduced.

Patient-reported outcome measures (PROMs) are useful tools to assess a patient’s quality of life from the patient’s perspective through a series of standardized and validated questionnaires [2]. Therefore, PROMs provide a unique opportunity in chronic disease management. Clinicians can use PROMs in routine patient care to monitor disease progression prior to patient visits to aid in effective treatment selection. Clinical manpower can be utilized more effectively by prioritizing patients with acute symptoms and reduce visits for patients with well-controlled symptoms, saving time and costs [6]. PROMs can also improve patient-physician communications by allowing patients to discuss their symptoms [7,8]. Additionally, providing feedback on PROM scores can encourage patients to track their symptoms regularly, and empower them with skills to manage their conditions better [8,9]. However, the effectiveness of feedback on PROMs with regards to effects on patient outcomes remain inconclusive [10].

Prior systematic reviews have ranged from evaluating the effects of PROMs feedback intervention in oncology [11,12], to evaluating the impact of integrating PROMs in routine pediatric clinical care [13]. A meta-analysis assessed the impact of using PROMs as a remote monitoring intervention in inflammatory arthritis [14]. However, they included studies that did not compare against conventional follow-up care. A scoping review identified the feasibility and utility of electronic PROMs and mobile health technology in rheumatic diseases; however, they did not assess patient outcomes [1]. Studies on rheumatic diseases have ranged from identifying commonly used PROMs in the routine care of rheumatoid arthritis [15], to assessing the impact of integrating PROMs use via qualitative methods from the perspectives of patients with rheumatoid arthritis, clinicians, and other staff [16,17]. To date, there is no comprehensive review that summarizes the effectiveness of PROMs use in self-symptom monitoring in rheumatic diseases.

Therefore, we aimed to provide an up-to-date synthesis of the effectiveness of PROMs use to self-monitor symptoms compared to conventional follow-up care in patients with rheumatic diseases. We also compared the effect of providing PROMs feedback to patients and/or healthcare professionals to a control group in which PROMs feedback was not provided.

## Methods

This study was conducted with reference to the PRISMA (Preferred Reporting Items for Systematic Reviews and Meta-Analyses) guidelines [18]. The protocol of this review was lodged in the OpenSci Framework [19]. (https://doi.org/10.17605/OSF.IO/ZU9XM).

### Search strategy

A systematic search was performed in October 2023 in PubMed, Embase, Cochrane Library Database and PsycINFO. The search terms were chosen in a way that any description that resembled or related to the use of PROMs to self-monitor symptoms in patients would be discovered by the search (S1 Table). Additional articles were identified by examining the reference lists of reviewed articles. The search was updated as of December 2024 and the start date for articles was unrestricted.

### Study selection

The study selection consisted of a two-phase process performed by two researchers. Two independent researchers (LO and PK) reviewed the database search results based on the titles and abstracts according to the inclusion and exclusion criteria. Articles that were unclear were carried forward into the full-text review. Full-text reports were sourced and independently evaluated by the same two reviewers (LO and PK). A third reviewer (ZH) was consulted in the event of a disagreement between the two researchers to independently assess the article.

Studies were eligible for inclusion when they used a PROM as an intervention for self-symptom monitoring in patients, with or without feedback to patients or healthcare professionals, compared with not using a PROM. Feedback received could be a summary of results or treatment advice based on the results of the PROM. Patients (18 years old or greater) with rheumatic diseases were included, and no specific care settings were in- or excluded. Randomized control trials, cohort studies, case control studies, case series and case reports were included. Studies were excluded if they were non-English language articles and abstract-only publications.

### Data extraction

Two reviewers (LO and PK) used a standardized data extraction form to independently extract data. The following study characteristics were extracted from each study: author, year, country, setting, study population, number of participants, study design, intervention, control, method in which PROMs are implemented and frequency (Table 1).

**Table 1:** Characteristics of included studies (N = 9).

Authors and year	Country	Setting	Study population	Sample size	Study Design	Intervention (I)	Control (C)	Method in which PROMs was implemented & frequency
Ackermans et al. 2018	Netherlands	Outpatient clinic of the orthopedic department	Osteoarthritis of the knee or hip	142 participants (70 in control; 72 in intervention)	Prospective cohort study	PROMs in the form of Web-based questionnaire measuring physical function and pain were administered, followed by a printout to feedback about their physical function and pain	Standard care as usual	Web-based PROMs questionnaires are completed before physician visits to rate their pain and rest assessment for the last week. Reports are printed and given to patients to discuss with physicians.
Gossec et al. 2018	France	Outpatient rheumatology care centre and one private practice clinic	Rheumatoid Arthritis	320 participants (161 in control; 159 in intervention)	Randomized Controlled Trial	Access without incentives to a web-based PROM after minimal training	Standard care as usual	Patients are not prompted to use the platform. If they recorded information, they could choose to share with their physicians.
Lee et al. 2021	USA	Outpatient clinic in a hospital	Rheumatoid Arthritis	191 participants (100 in control; 91 in intervention)	Randomized controlled trial	Smartphone app containing PROMs with a clinical case manager	Clinical case manager alone	App notifies patients to complete PROMs daily; clinical case manager only reviews response when they receive a flare notification and notifies the physician.
Li et al. 2023	China	Outpatient clinic in a hospital	Rheumatoid Arthritis	2204 participants (1098 in control; 1099 in intervention)	Randomized controlled trial	Smartphone app with PROMs	Standard care as usual	Patients complete PROMs every month
Pers et al. 2021	France	Outpatient clinic in a hospital	Rheumatoid Arthritis	94 participants (44 in control; 45 in intervention)	Randomized controlled trial	Smartphone app with PROMs	Standard care as usual	A clinical care manager sends the patients emails to complete PROMs weekly. The clinical care manager is automatically notified of problems experienced by the patient and can choose to inform the rheumatologist in-charge.
Salaffi et al. 2016	Italy	Outpatient clinic	Early Rheumatoid Arthritis	41 participants (20 in control; 21 in intervention)	Randomized Controlled Trial	Web-based PROM to tele-monitor symptoms with monthly visits.	Standard care of face-to-face visits	Frequency of PROMs data collection is not mentioned. A clinical case manager is notified of suggestions to contact physicians when a patient’s scores deteriorate.
Seppen et al. 2022	Netherlands	Outpatient rheumatology care centre	Rheumatoid Arthritis	103 participants (53 in control; 50 in intervention)	Randomized Controlled Trial	Self‐monitoring using a smartphone app (RAPID-3 questionnaires) with a single preplanned consultation at the end of the trial period.	Standard care as usual	App notified patients weekly to complete the PROMs questionnaire
Shaw et al. 2021	Switzerland	Outpatient clinic in hospitals, private rheumatology clinics	Rheumatoid arthritis, Axial spondylarthritis, Psoriatic arthritis	2111 participants (1799 non-app users, 150 in app-only group, 162 in app & discussion group)	Prospective Cohort Study	**Intervention 1:** SCQM app use containing PROMs with no discussion with physicians. **Intervention 2**: SCQM app use containing PROMs with discussion with physicians. *(In* ***Jan 2019****: iDialog was replaced with an updated app renamed as MySCQM.)*	No SCQM app use	iDialog solicited monthly data entries from users with the RADAI-5. MySCQM app allows patients to choose monthly or weekly data entry for PsA patients
Solomon et al. 2024	USA	Outpatient academic medical centre and rheumatology clinics	Rheumatoid Arthritis	300 participants (150 as matched controls; 150 in intervention)	Prospective Cohort study	PROMS in the form of an app integrated into EHR	No PROMs app	Four validated PROM short forms were delivered through the app with SMS push notification reminders (one PROM every 48 hours). The app was integrated into the EHR, allowing rheumatologists to easily view the PROM data.

Abbreviations: EHR (Electronic Health Record), PROMs (Patient Reported Outcome Measures), PROs (Patient Reported Outcomes), RAPID-3 (Routine Assessment of Patient Index Data-3), RADAI-5 (Rheumatoid Arthritis Disease Activity Index-5), SCQM (Swiss Clinical Quality Management in Rheumatic Diseases). PsA (Psoriatic Arthritis).

### Risk of bias

Risk of bias evaluation of all included studies was performed independently by the same two reviewers using the Cochrane Collaboration Risk of Bias (ROB) Tool for randomized controlled trials [20]. The Risk of Bias in Non-randomized Studies of Interventions (ROBINS-I) was used to assess non-randomized studies [21]. These tools are crucial in evaluating the quality of evidence by identifying potential bias and limitations of the included studies which may influence the interpretation of findings in this systematic review.

### Data synthesis and analysis

Two independent reviewers (LO and PK) performed data synthesis and analysis. Extracted outcomes and experiences were synthesized in a narrative matter and categorized into one of five categories: health-related quality of life (HRQoL), symptoms/morbidity, patient perception (level of understanding of their disease, patient satisfaction regarding care), process indicators (number of topics discussed, management/treatment actions, patient-doctor communication), and clinic outcomes (reduction in number of clinic visits). Subsequently, we compared the effect of providing feedback via PROMs to a control group in which PROMs feedback was not provided.

## Results

### Study characteristics

**Fig 1:**
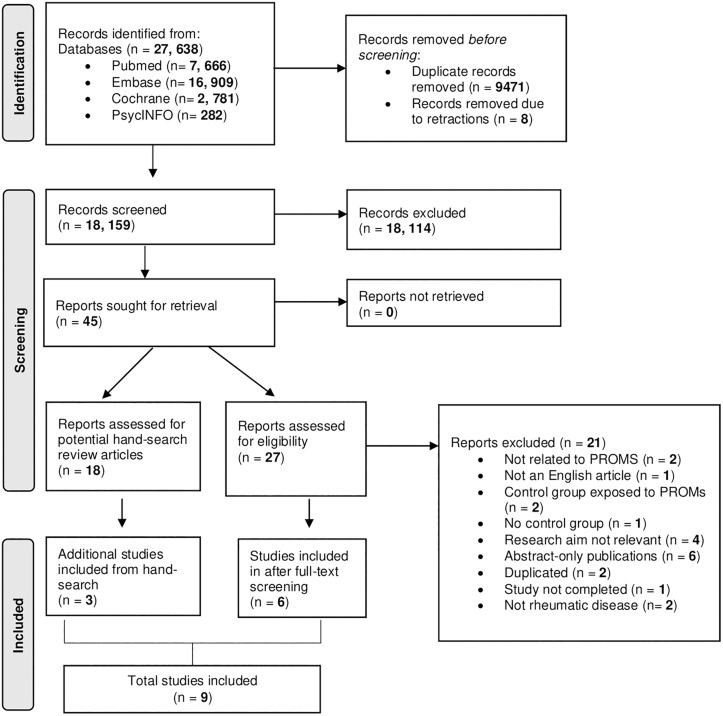
PRISMA flow diagram of included studies.

After removal of duplicates, 18,159 references were identified through the initial search. After screening the titles and abstracts, forty-five articles were eligible for full-text review. During full-text screening, 18 were identified as potential review articles for hand-searching. 3 additional studies were included by searching the reference lists of previously published literature reviews [22–24]. Out of 27 primary literature, 6 studies met the inclusion criteria. As such, a total of 9 articles were included in the final analysis (Fig 1).

Of the included studies, 6 (67%) were randomized controlled trials while 3 (33%) were cohort studies. All the studies were conducted in an outpatient setting. Patients with various rheumatic conditions such as rheumatoid arthritis, osteoarthritis, axial spondylarthritis and psoriatic arthritis participated in the studies. The full characteristics of the studies are in Table 1.

Majority of the studies were conducted in Europe (n = 6). One study was conducted in China, and two were conducted in the USA. A range of PROMs were used in the studies such as the Rheumatoid Arthritis Disease Activity Index-5 score, Bath Ankylosing Spondylitis Disease Activity Index score, Treatment Satisfaction Questionnaire for Medication, Rheumatoid Arthritis Impact of Disease score, PROMs Hip disability and Osteoarthritis Outcome Score–Physical function short form and Knee disability and Osteoarthritis Outcome Score–Physical function short form. The Routine Assessment of Patient Index Data-3 (n = 5) was the most used PROMs.

### Risk of bias

S2 Table summarizes the risk of bias of RCT studies using the Cochrane RoB Tool [20]. Only one study was rated to be at high risk of bias [25]. Bias from the randomization process was high as the method of randomization was not stated. The extent of missing outcome data was not reported, and sensitivity analyses were not conducted to correct for potential bias [25]. Additionally, the investigators were not blinded.

S3 Table summarizes the risk of bias of non-randomized studies using the ROBINS-I tool [21]. Two studies were rated to be of moderate risk of bias [26,27], while one study was rated to be of low risk [28]. The moderate risk of confounding in the two studies was attributed to baseline differences between the groups. The study by Shaw et al was rated to be of moderate risk for missing data as it lacked measures of disease activity for patients with undifferentiated arthritis [27]. Furthermore, information on the proportion of missing outcome data was missing between the intervention groups. Another study lacked information on the extent of missing data [26].

### PROM as intervention compared with not using a PROM

All nine included studies compared the use of a PROM as an intervention to no PROM use as a control [22–27,29,30]. In six studies, PROM scores were provided to both the healthcare professional and the patient [22–24,27–30]. Feedback in the form of PROM scores was provided only to the patient in two studies [25,26], and scores were only available to the healthcare professional if the patient proactively shared them [25]. Lastly, in one study, PROM scores were only provided to the healthcare professional [28] (Table 2).

**Table 2:** Main findings comparing the use of PROMs as intervention to not using a PROM.

Author and year	Survival/mortality	Symptoms/Morbidity	Health-Related Quality of Life (HRQoL)	Patient Perception	Shared Decision Making	Process indicators	Clinic outcomes	Others
Ackermans et al. 2018	NR	NR	NR	NR	NR	NR	NR	A higher level of self-efficacy was associated with a higher level of patient empowerment (coefficient = 0.40, p = 0.001) and satisfaction (coefficient = 0.34, p = 0.001), but age, education level, marital status, etc, were not associated.
Gossec et al. 2018	NR	RAID changes did not differ between groups; both IG and CG saw an improvement in RAID score at 12 months; further improvement was seen in the coping component of the RAID for the IG although differences were not statistically significant.	NR	Patient satisfaction was high among patients in the IG who still followed up: mean: 1.5 (SD:1.5), median 1 [on a scale of 0–10, with 0 being total satisfaction] Changes in perceived quality of care were different between the groups. Mean (SD) changes in quality-of-care NRS from baseline to 12 months were 8.2 (1.7) to 8.3 (1.6) vs 8.2 (1.6) to 7.8 (1.9) in the IG vs CG (p = 0.02).	NR	A small improvement in patient-physician interactions score was seen in patients in the IG compared to the CG, for whom a concurrent decline in scores was observed, leading to a significant difference. Mean (SD) changes in PEPPI from baseline to 12 months were 38.6(8.2) to 39.2 (8.0) vs 39.7(7.3) to 38.8 (8.0) in IG vs CG (p = 0.01).	NR	NR
Lee et al. 2021	NR	Among 91 patients in IG, mean scores for RADAI-5 decreased from 3.0 at baseline to 2.7 at 24 weeks. Mean PROMIS fatigue scores decreased from 52.7 to 47.6.	NR	58% of the IG thought that the combination of the intervention with a clinical care coordinator was most helpful whereas 30% thought the app was most helpful.	NR	11 physicians responded to an exit survey. 27% agreed that the app led to earlier changes in medications whereas 45% were neutral/strongly disagreed that the app improved communication. 9% felt that the app increased their workflow.	NR	NR
Li et al. 2023	NR	At month 6, there was a higher rate of patients with a DAS28-CRP score of 3.2 or less, as determined by the modified ITT analysis after multiple imputation. 71.0% (780 of 1099) in IG vs 64.5% (708 of 1098) in the CG.	NR	NR	NR	NR	NR	NR
Pers et al. 2021	NR	No differences between IG and CG were found in DAS28, RAPID-3, HAQ scores – the percentage of patients in remission and low disease activity, as well as the average RAPID-3 score at six months, were similar in both groups. Mean change in the DAS28 score over the 6 months of the study did not differ significantly between the groups: –1.48 (s.e.m. = 0.16) in the CG and –1.37 (s.e.m. = 0.16) in the IG (P-value = 0.63). There was a statistical difference with a lower HAQ score in the IG at six months [0.56 (0.46) *vs* 0.78 (0.59); *P* = 0.043].	There were significant improvements in several domains in the IG after six months. Patients reported a better quality of life, in particular for the RP and the RE scores. When considering changes in delta SF-12 from baseline to six months, there was greater improvement in the IG group for PF and RP scores. All SF-12 domains showed a greater increase in quality of life in the IG.	The IG group had a high satisfaction rate with all facets of the application (technical, safety, availability). 80% were eager to continue using the application.	NR	NR	4.4% (n = 2) of the patients in the IG vs 86.4% in the CG had at least 2 physical visits during the six-month study (P < 0.01), resulting in a significantly lower number of intermediate physical visits in the IG group compared to the CG group. Conversely, the number of phone-call visits was significantly higher in the IG group. (P < 0.01) Interestingly, the total number of visits found no statistical significance between the IG and CG group.	NR
Salaffi et al. 2016	NR	Higher percentage of patients in IG achieved CDAI remission versus the CG. (38.1% vs 25% at year 1, P < 0.01). Time to achieve remission (CDAI <2.8) was significantly shorter in IG than in CG, with a median of 20 weeks versus a median over 36 weeks (P < 0.001).	NR	High satisfaction among the vast majority in IG, with an overall average of 4.28 out of 5 (where 1 = not at all, 5 = enormously) in response to the intervention.	NR	NR	NR	NR
Seppen et al. 2022	NR	The DAS28‐ESR slightly increased in both groups (ΔDAS28‐ESR in IG was 0.27 versus 0.35 in CG). Noninferiority was established, as the 95% CI of the mean difference in DAS28‐ESR between the groups was within the noninferiority limit of −0.04 in favor of the IG (95% CI −0.39, 0.30), adjusted for baseline DAS28‐ESR (no significant confounders). No significant differences between groups in RAPID3 scores at 12 months.	NR	Satisfaction with health care was high in both groups and not statistically different.	NR	No significant differences between groups in patient-physician interaction at 12 months.	After 12 months, the number of rheumatologist telephone consultations and outpatient clinic visits was significantly lower in the IG, with a total visit rate ratio of 0.6 (95% CI 0.47, 0.80) relative to the total number of visits in the CG (P < 0.001). The total number of outpatient visits with nurses was also lower in the IG. (0.4 in IG vs 0.8 in the CG). (P = 0.03)	No significant differences between groups in medication adherence or self-management at 12 months.
Shaw et al. 2021	NR	Differences in adjusted disease management outcomes between IG and CG were not statistically significant. (P = 0.05). Patients with higher disease activity at baseline were less likely to achieve low disease activity at the last SCQM visit (OR 0.33, 95% CI 0.26 to 0.42), more likely to have improved disease activity by the last SCQM visit (OR 1.20, 95% CI 1.01 to 1.43) and more likely to intensify therapy in the last 6 months of follow-up (OR 1.34, 95% CI 1.09 to 1.65).	NR	NR	In adjusted analysis, the intervention+ feedback group was more likely to be satisfied with SDM (OR 1.66, 95% CI 1.14 to 2.42) and with physician disease tracking (OR 2.00, 95% CI 1.30 to 3.09) compared with CG, whereas the intervention-only group had similar levels of satisfaction with these outcomes compared with CG.	NR	NR	NR
Solomon et al. 2024	NR	There were slightly fewer flares reported in the application users (25%) than matched nonusers (28.5%; P value from mixed effect logistic regression = 0.44 for the between-group difference) In terms of patient reported outcome results, individual patient’s symptoms varied, but the median values were stable throughout the follow-up years	NR	NR	NR	NR	Both groups demonstrated similar monthly visit volumes. There was no statistical difference in the differences between the groups. (−2.7 visits, 95% CI –9.3 to 4.0). In the intervention cohort, the estimated monthly visit volume in the year before using the application was 31.2 (95% CI 27.3–33.6) compared to 30.4% (95% CI 27.3–33.6) in the controls for the same period.	NR

Abbreviations: NR (not reported), IG (intervention group), CG (control group), SDM (Shared Decision Making), RAPID-3 (Routine Assessment of Patient Index Data-3), DAS28 (Disease Activity Score 28), DAS28-ESR (Disease Activity Score 28-Erythrocyte Sedimentation Rate), RAID (Rheumatoid Arthritis Impact of Disease), CDAI (Clinical Disease Activity Index), PEPPI (Perceived Efficacy in Patient-Physician Interactions), RADAI-5 (Rheumatoid Arthritis Disease Activity Index-5), DAS28-CRP (Disease Activity Score 28-C-Reactive Protein), RP (role limitations due to physical health problems); PF(physical functioning), SF-12(12-item Short Form Health Survey), HAQ (Health Assessment Questionnaire), PROM (Patient Reported Outcome Measure) s.e.m. (standard error of mean), SD (standard deviation), SCQM (Swiss Clinical Quality Management in Rheumatic Diseases).

### Survival/mortality

None of the studies reported survival as an outcome.

### Symptoms and morbidity

Symptoms control/morbidity was the most assessed outcome in evaluating PROM interventions.

In terms of morbidity, three studies did not identify significant differences between the intervention and the control group [25,27]. Solomon et al [28], found that the intervention group had slightly fewer flares reported (25%) compared to the control (28.5%). In Gossec et al [25] the Rheumatic Arthritis Impact of Disease (RAID) changes did not differ between both groups, but both saw improvements in RAID scores at 12 months in rheumatoid arthritis patients. However, Shaw et al [27] found that for patients with higher disease activity at baseline, they were less likely to achieve low disease activity by their last PROM visit but were likely to have improved disease activity clinically as determined by the physician. The study looked at patients with rheumatoid arthritis, axial spondylarthritis and psoriatic arthritis.

Conversely, Seppen et al conducted a non-inferiority trial and established non-inferiority between the intervention and control groups [24]. The mean difference in the Disease Activity Score-28 for Rheumatoid Arthritis with Erythrocyte Sedimentation Rate was within the noninferiority limit of –0.04, in favor of the intervention group (95% CI –0.39, 0.30). This resulted in a 38% decrease in rheumatologist consultations in rheumatoid arthritis patients with low disease activity. Additionally, four studies showed an improvement in symptom control in the intervention group [22,23,29,30]. Salaffi et al found that a higher percentage of patients in the intervention achieved remission (P < 0.01), and the time to achieve remission was significantly shorter [22]. Three randomized controlled studies reported that the intervention group had lower disease scores [23,29,30].

### HRQoL

Pers et al reported that the intervention group had significant improvements in HRQoL with the use of PROMs, with all Short Form-12 domains showing a greater increase [23].

### Patient perception

Five studies reported on patient perception regarding patient-physician communication, quality of care, and the app used to administer PROMs [22–25,30].

While four of the studies did not report whether the results were statistically significant, high patient satisfaction was observed in the intervention groups in these categories. Only one study reported that there was no statistically significant difference between the intervention and control groups in their satisfaction towards health care [24].

### Shared Decision Making (SDM)

Shaw et al reported that the PROMs use with feedback group was more likely to be satisfied with SDM and physician disease tracking, whereas the PROMs-only group had similar levels of satisfaction as compared to the non-PROM users [27].

### Process indicators

Two studies reported on patient-physician interaction with mixed results [24,25]. Seppen et al [24] did not find any significant differences between the groups. However, Gossec et al [25] reported that there was a significant difference between the groups (intervention vs control), with a small improvement in the patient-physician interaction score, measured using the Perceived Efficacy in Patient-Physician Interactions-5 score.

Lee et al [30] interviewed the physicians providing care to the participants with mixed responses. Of the responses collected, 27% felt that collecting information on PROMs via an app led to earlier medication changes, 45% felt that it did not improve communication and 9% felt that it increased their workflow instead.

### Clinic outcomes

Three studies investigated the change in clinic visits [23,24]. In the randomized controlled trial by Seppen et al [24], the number of telephone consultations and outpatient clinic visits in the intervention group was significantly lower. The total visit rate ratio was lower compared to the control group (P < 0.01). Another study reported that while the total number of physical visits reduced in the intervention group, there was an increase in phone-call visits [23]. This resulted in no statistical significance observed in terms of total number of visits (i.e., sum of physical and phone-call visit) between the intervention and control groups. Solomon et al [28] compared the reduction in monthly visits volume between the intervention and control group, regardless of whether it was before the PROMs was used, or in the same year that the PROMs was used. There was no significant difference in the differences between the two groups.

### Others

Ackermans et al [26] found that patients with a higher level of self-efficacy were associated with a higher level of patient empowerment and satisfaction.

In the randomized controlled trial by Seppen et al [24], no significant differences were observed between groups in medication adherence or self-management.

### PROMs with feedback to patients and/or healthcare professionals in an intervention group versus PROMs without feedback in a control group

Two studies compared the use of PROMs as intervention with feedback provided to patients, against a control group in which PROMs were also used, but feedback regarding PROM scores was not provided [26,27] (Table 3). In Shaw et al [27], there was an additional intervention group where feedback was provided to both the patient and the physician.

**Table 3:** Findings comparing PROMs with feedback to patients and/or healthcare professionals as an intervention versus PROMs without feedback in a control.

Author and year	Survival/mortality	Symptoms/Morbidity	Health-Related Quality of Life (HRQoL)	Patient Perception	Process Indicators	Others
Ackermans et al. 2018	NR	NR	NR	No differences between the group given PROM feedback and the group that did not receive such feedback in terms of satisfaction scores (8.6 ± 1.4 versus 8.8 ± 1.2; mean difference −0.19; p = 0.39) and patient-physician relationship (4.3 ± 0.69 versus 4.4 ± 0.56; mean difference −0.09; p = 0.41).	NR	NR
Shaw et al. 2021	NR	NR	NR	App+feedback group was more satisfied with apps compared with those in the app-only group. A greater percentage of users in the app+feedback group found the apps easy to use (72% vs 92%, p < 0.001) and understand (83% vs 95%, p = 0.004), and were more likely to recommend the apps to others (41% vs 72%, p < 0.001).	NR	NR

Abbreviations: NR (Not reported).

### Patient perception

One study did not find significant differences between the two groups in terms of their satisfaction scores or in the patients’ perception of the patient-physician relationship [26].

However, Shaw et al [27] found that the intervention group was more satisfied with using an app to monitor their symptoms. The app was easy to use to document their symptoms, and charts were used to reflect PROMs scores as feedback. This made it easier for the patients to understand their symptoms and discuss their results with their healthcare professional.

## Discussion

This systematic review summarizes the evidence of using PROMs in clinical settings. To the best of our knowledge, this is the first systematic review that evaluates the effectiveness of PROMs in the setting of rheumatic diseases, which was the first aim of this paper. We did not find evidence that PROMs use could affect survival and mortality in patients with rheumatic diseases. However, our study revealed that PROMs may improve symptom control, HRQoL, SDM, patient satisfaction and reduce clinic visits.

Our findings were largely consistent with other studies that demonstrated improved patient outcomes in other diseases such as symptom control, HRQoL, SDM, patient satisfaction and reduced clinic visits with the use of PROMs. This is because research has proven that regular collection and symptom monitoring using PROMs improves symptom management [7,12]. It allows clinicians to detect complications quickly, intervene early in patients who require treatment modifications, and therefore take immediate action to reduce the symptom burden on the patient [6]. This contributes to better symptom control and thus, patients can report an improved HRQoL and greater satisfaction. Frequent symptom monitoring also allows clinicians to identify stable patients and thus reduce their appointment frequencies, resulting in fewer visits [6].

While we observed improvements in HRQoL and reduction in clinic visits, an interesting finding from Pers et al [23] was the addition of a clinical case manager (CCM) in addition to PROMs use in the intervention group. Hence, the positive results (e.g., improvement in HQRoL, reduction in physical consultation and the number of trips to the hospital) could be confounded by the additional support of the CCM [23]. Furthermore, the reduction in physical visits may have been due to more phone-call visits being conducted because patients needed reassurance for the lack of physical visits. Another intriguing finding from Solomon et al [28] was that the monthly visit volume remained similar in both groups before and after using the PROM application in the intervention. This could be attributed to factors such as that PROMs are not designed to change visit frequency but are instead validated measures of symptom control; and there were physicians’ concerns about longer time intervals between visits. Longer time intervals for follow-up would require greater laboratory monitoring and patient education [28].

The lack of findings on survival and mortality outcomes differs from other systematic reviews that found that PROMs could increase the survival rate of patients with cancer [11,12]. This could be because patients with rheumatic diseases tend to have longer life expectancy and mortality is likely due to other comorbidities as compared to other chronic diseases like cancer [31]. Better management of rheumatic diseases can help to reduce mortality.

In the second aim of the review, we investigated the effect of providing feedback via an app on PROMs use to patients and/or health care professionals. One study found that feedback increased patient satisfaction [27]. This was attributed to physician feedback providing a platform to talk about their symptoms, facilitating SDM and hence improving physician-patient interactions. Our finding is similar to Graupner et al who also revealed that receiving feedback resulted in better symptom control, higher patient satisfaction and improved patient-physician communication during cancer care [11]. Conversely, another study reported that there was no difference regardless of whether feedback was provided [26]. This could be due to the feedback being provided only over a single visit and the study period was relatively short, hence it might not be conclusive to dismiss its effectiveness. More studies are needed to determine if regular feedback provided over a longer period could improve patient outcomes and satisfaction.

Our study possesses several strengths. We conducted a comprehensive search across four databases and used sensitive search filters to capture as many potentially relevant articles as possible. The rigor of the study was established using the PRISMA statement, which is well regarded as a consensus-based standard.

However, our study also has limitations. The quality appraisal revealed that three of the included studies were of moderate to high risk of bias. Missing outcome data can raise issues to the validity and generalizability of these studies [25–27]. Secondly, the selection and evaluation of articles by the reviewers were subjective in nature and may be prone to judgment bias. Nevertheless, the requirement by PRISMA to have two independent reviewers and the need for a third reviewer to reach a consensus in the case of any discrepancies has helped reduce the risk of judgment bias. Thirdly, the data synthesis was narrative due to large variety across all studies such as PROMs types, evaluated outcomes, feedback given to patient, and follow-up time. It was therefore difficult to perform any type of quantitative synthesis or compare individual studies to each other. Fourthly, as our patient sample primarily consisted of rheumatoid arthritis patients, there was a lack of diversity which limits the generalizability of our findings. Despite this, even though patients with SLE may experience different symptoms and lab results that do not always align with each other, PROMs are still useful for symptom monitoring as well. For example, SLE patients can experience persistent fatigue and pain that affects their HRQoL although their condition may be in remission [32]. However, physicians may not associate these symptoms with SLE and thus may not address these unmet medical needs during consultations. In systemic sclerosis patients, initial symptoms such as fatigue tend to be non-specific and Raynaud’s phenomenon is typically episodic and can be difficult to measure using objective methods. As a result, this often leads to delays in diagnosis and treatment [33]. Fifthly, we acknowledge that the analysis of the effectiveness providing feedback via an app on PROMs would be more comprehensive if the feedback on PROMs was classified through a conceptual framework. However, there is no suitable framework in the current literature that allows for the classification of feedback on PROMs. Lastly, PROMs differ in structure, frequency, administration mode, and associated interventions. For example, even when the PROMs administered to patients is identical, the method of administration may differ. Both studies administered RAPID-3 to patients, but Seppen et al utilized a smartphone application while Pers et al utilized an email-prompted web-based questionnaire [23,24]. The heterogeneity in the implementation of PROMs may result in unexpected differences in the effectiveness of PROMs in symptom monitoring, Nevertheless, despite these limitations, our review still offers valuable insights into the effectiveness of PROMs in symptom monitoring.

Future research could delve into the potential implication of integrating PROMs into clinical practice. With information on patient experience in clinical care and how it influences treatment choices, it aims to enhance personalized care for patients. Additionally, investigating the impact of PROMs on healthcare resource utilization seeks to make healthcare more accessible and affordable. Further studies could also examine the effectiveness of PROMs across a wider range of rheumatic diseases, to support a broader implementation of PROMs into clinical practice.

In conclusion, we found that PROMs use can improve symptom control, HRQoL and reduce clinic visits in rheumatic diseases, particularly in rheumatoid arthritis. There was conflicting evidence on patient satisfaction when providing feedback to the patient and/or healthcare professional. However, providing feedback on PROMs use in rheumatic diseases can facilitate shared decision making. This review has provided evidence that the use of PROMs in clinical care may improve patient outcomes in patients with rheumatic diseases.

## Supporting information

S1 Table Search strategy. (DOCX)

S2 Table Cochrane risk of bias of individual studies. (DOCX)

S3 Table Risk of bias in non-randomized studies of intervention of individual studies. (DOCX)

S4 Table List of studies. (XLSX)

S5 Table Glossary. (DOCX)

S6 Table PRISMA checklist. (DOCX)

S7 Table PRISMA abstract checklist. (DOCX)
