# Supplementary material for: A systematic review of the effectiveness of self-symptoms monitoring with Patient Reported Outcome Measures in rheumatic disease patients
Source: PLoS One. 2025 Dec 30;20(12):e0338935. doi: 10.1371/journal.pone.0338935 (PMC12753051; doi:10.1371/journal.pone.0338935)
Supplement: S1 Table — (DOCX) [file pone.0338935.s001.docx]

**Supplementary Table S1. Search Strategy**

**PubMed**

| **Components** | **Keywords** |
| --- | --- |
| **Population**  (Disease terms) | 1. "Arthritis" [MeSH] OR "Lupus Erythematosus, Systemic" [MeSH] OR "Vasculitis" [MeSH] OR "Myositis" [MeSH] OR "Scleroderma, Systemic" [MeSH] OR "Fibromyalgia" [MeSH] 2. "rheumatoid arthritis" [tiab] OR "psoriatic arthritis" [tiab] OR "reactive arthritis" [tiab] OR "osteoarthritis" [tiab] OR "spondyloar*" [tiab] OR "ankylosing spondy*" [tiab] OR "axial spondylarthr*" [tiab] OR "Systemic Lupus Erythematosus" [tiab] OR "Gout" [tiab] OR "Lupus" [tiab] OR "Vasculitis" [tiab] OR "Myositis"[tiab] OR "Dermatomyositis"[tiab] OR "Scleroderma" [tiab] OR "Fibromyalgia" [tiab] OR "Sjogren's Syndrome" [tiab] |
| **Intervention**  (PROMs keywords) | 1. Patient reported outcome measures [MeSH] 2. “Patient reported outcome measure*” [tiab] 3. “Patient reported outcome*” [tiab] 4. “Patient outcome assessment*”[tiab] 5. “patient reported treatment outcome* “[tiab]. 6. (PROs or PROM or PROMs or PROMIS) [tiab] 7. “self-report* measure*” [tiab] 8. “self-report* outcome*” [tiab] |
|  | 1. #1 OR #2 2. #3 OR #4 OR #5 OR #6 OR #7 OR #8 OR #9 OR #10 3. #11 AND #12 |

**PsycINFO**

| **Components** | **Keywords** |
| --- | --- |
| **Population**  (Disease terms) | 1. arthritis/ or rheumatoid arthritis/ or Fibromyalgia/ 2. "rheumatoid arthritis" [tiab] OR "psoriatic arthritis" [tiab] OR "reactive arthritis" [tiab] OR "osteoarthritis" [tiab] OR "spondyloar*" [tiab] OR "ankylosing spondy*" [tiab] OR "axial spondylarthr*" [tiab] OR "Systemic Lupus Erythematosus" [tiab] OR "Gout" [tiab] OR "Lupus" [tiab] OR "Vasculitis" [tiab] OR "Myositis"[tiab] OR "Dermatomyositis"[tiab] OR "Scleroderma" [tiab] OR "Fibromyalgia" [tiab] OR "Sjogren's Syndrome" [tiab] |
| **Intervention**  (PROMs keywords) | 1. Patient reported outcome measures [MeSH] 2. “Patient reported outcome measure*” [tiab] 3. “Patient reported outcome*” [tiab] 4. “Patient outcome assessment*”[tiab] 5. “patient reported treatment outcome*” [tiab]. 6. (PROs or PROM or PROMs or PROMIS) [tiab] 7. “self-report* measure*” [tiab] 8. “self-report* outcome*” [tiab] |
|  | 1. #1 OR #2 2. #3 OR #4 OR #5 OR #6 OR #7 OR #8 OR #9 OR #10 3. #11 AND #12 |

**Embase**

| **Components** | **Keywords** |
| --- | --- |
| Population  (Disease terms) | 1. arthritis'/exp OR 'systemic lupus erythematosus'/exp OR 'vasculitis'/exp OR 'myositis'/exp OR 'systemic sclerosis'/exp OR 'fibromyalgia'/exp OR 'sjoegren syndrome'/exp 2. 'rheumatoid arthritis':ab,ti OR 'psoriatic arthritis':ab,ti OR 'reactive arthritis':ab,ti OR osteoarthritis:ab,ti OR spondyloar*:ab,ti OR 'ankylosing spondy*':ab,ti OR 'axial spondylarthr*':ab,ti OR 'systemic lupus erythematosus':ab,ti OR gout:ab,ti OR vasculitis:ab,ti OR myositis:ab,ti OR dermatomyositis:ab,ti OR scleroderma:ab,ti OR fibromyalgia:ab,ti OR 'sjoegren syndrome':ab,ti |
| **Intervention**  (PROMs keywords) | 1. 'Patient-reported outcome'/exp 2. 'Patient reported outcome measure*':ab,ti 3. 'Patient reported outcome*':ab,ti 4. 'Patient outcome assessment*':ti,ab 5. 'Patient reported treatment outcome*':ti,ab 6. pros:ti,ab OR 'prom':ab,ti OR 'proms':ab,ti OR 'promis':ti,ab 7. 'self-report* measure*':ti,ab 8. 'self-report* outcome*':ti,ab |
|  | 1. #1 OR #2 2. #3 OR #4 OR #5 OR #6 OR #7 OR #8 OR #9 OR #10 3. #11 AND #12 |

**Cochrane**

| **Components** | **Keywords** |
| --- | --- |
| **Population**  (Disease terms) | 1. "Arthritis" [MeSH] OR "Lupus Erythematosus, Systemic" [MeSH] OR "Vasculitis" [MeSH] OR "Myositis" [MeSH] OR "Scleroderma, Systemic" [MeSH] OR "Fibromyalgia" [MeSH] 2. spondyloar*:ti,ab OR ankylosing NEXT spondy*:ti,ab OR axial NEXT spondylarthr*:ti,ab 3. "psoriatic arthritis":ti,ab OR "osteoarthritis":ti,ab OR "rheumatoid arthritis":ti,ab OR “reactive arthritis”:ti,ab 4. gout:ti,ab OR "systemic lupus erythematosus":ti,ab OR lupus:ti,ab OR vasculitis:ti,ab OR myositis:ti,ab OR dermatomyositis:ti,ab 5. scleroderma:ti,ab OR fibromyalgia:ti,ab or 'Sjogren's Syndrome':ti,ab |
| **Intervention**  (PROMs keywords) | 1. [Patient Reported Outcome Measures] MeSH 2. "Patient reported" NEXT outcome*:ti,ab 3. "Patient reported outcome" NEXT measure*:ti,ab 4. "Patient outcome" NEXT assessment*:ti,ab 5. "Patient reported treatment" NEXT outcome:ti,ab 6. self-report* NEXT measure*:ti,ab 7. self-report* NEXT outcome*:ti,ab 8. PRO:ti,ab OR PROM:ti,ab OR "PROMs":ti,ab OR "PROMIS":ti,ab |
|  | 1. {or #1-#5} 2. {or #6-#13} 3. #14 AND #15 |
