# Supplementary figures and images for: A systematic review of the effectiveness of self-symptoms monitoring with Patient Reported Outcome Measures in rheumatic disease patients
Source: PLoS One. 2025 Dec 30;20(12):e0338935. doi: 10.1371/journal.pone.0338935 (PMC12753051; doi:10.1371/journal.pone.0338935)

**Supplementary Table S2.** Cochrane Risk of Bias of individual studies


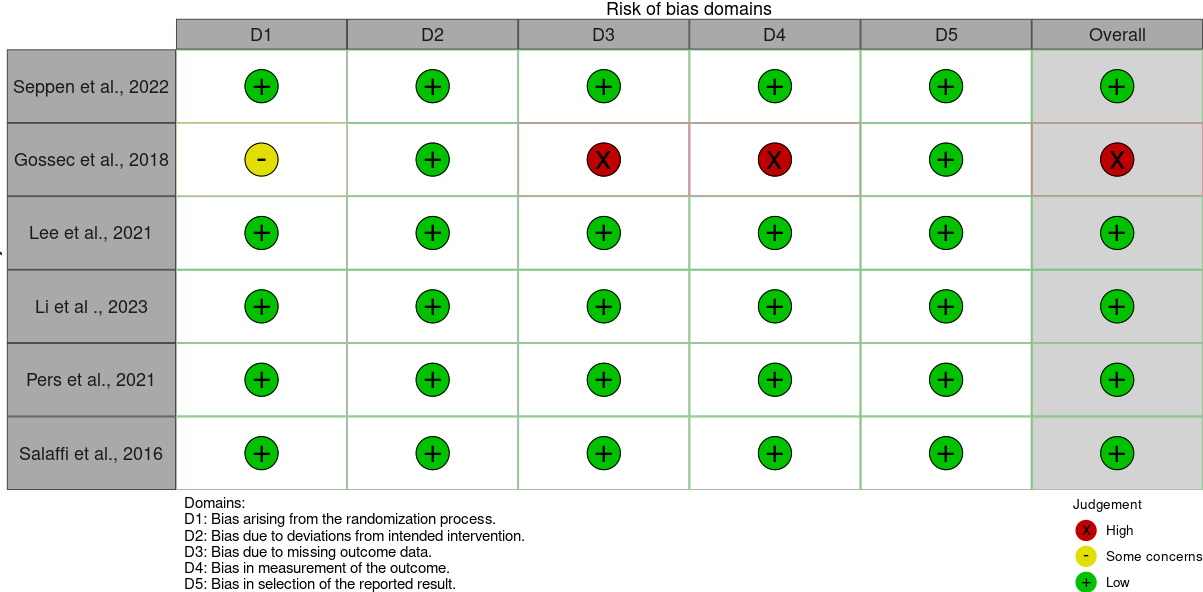

Supplement: S2 Table — (DOCX) [file pone.0338935.s002.docx]
