# Supplementary material for: A systematic review of the effectiveness of self-symptoms monitoring with Patient Reported Outcome Measures in rheumatic disease patients
Source: PLoS One. 2025 Dec 30;20(12):e0338935. doi: 10.1371/journal.pone.0338935 (PMC12753051; doi:10.1371/journal.pone.0338935)

**Supplementary Table S3.** Risk Of Bias In Non-randomized Studies of Intervention of individual studies


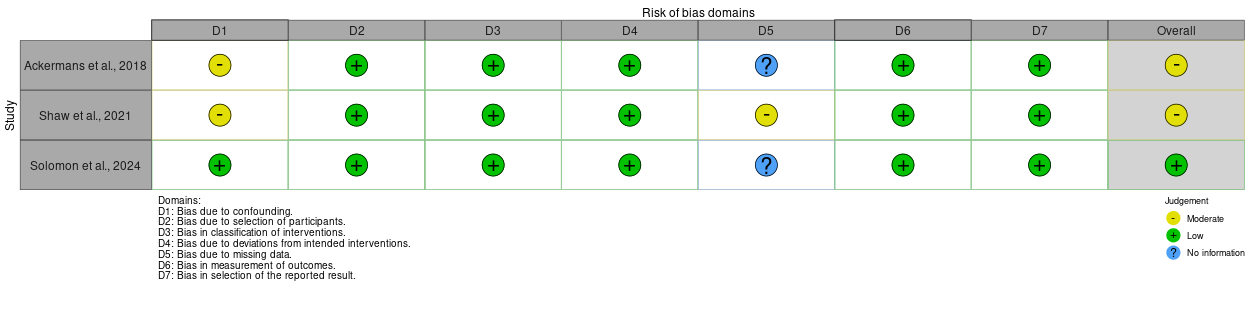

Supplement: S3 Table — (DOCX) [file pone.0338935.s003.docx]
