# Supplementary material for: A systematic review of the effectiveness of self-symptoms monitoring with Patient Reported Outcome Measures in rheumatic disease patients
Source: PLoS One. 2025 Dec 30;20(12):e0338935. doi: 10.1371/journal.pone.0338935 (PMC12753051; doi:10.1371/journal.pone.0338935)
Supplement: S5 Table — (DOCX) [file pone.0338935.s005.docx]

**Supplementary Table S5. Glossary**

| **Term** | **Definitions** |
| --- | --- |
| **Patient-reported outcome measures (PROMs)** | PROMs questionnaires that collect health outcomes directly from the people who experience them.   - Examples include Rheumatoid Arthritis Disease Activity Index (RADAI) |
| **Patient reported outcomes (PRO)** | PROs are measured using PROMs and rated on a scale.   - Examples include pain, quality of life, fatigue and physical function. |
| **Feedback** | Feedback received could be a summary of results or treatment advice based on the results of the PROMs. The summary of results/treatment advice could be provided to patients and/or to healthcare professionals. |
| **Intervention** | Intervention group refers to the group in which PROMs are used, regardless of whether feedback was provided. |
| **Control** | Control group refers to the group, in which no PROMs are used |
